# Analysis of oral microbiome characteristics and their correlation with oral health diseases

**DOI:** 10.1097/MD.0000000000048600

**Published:** 2026-05-08

**Authors:** Yujie Nie, Xiaohui Lin

**Affiliations:** a Guangdong Provincial People’s Hospital’s Nanhai Hospital, Foshan, Guangdong, China.

**Keywords:** dental caries, lifestyle factors, microbial diversity, oral health, oral microbiome, periodontitis

## Abstract

To investigate the relationship between oral microbiome characteristics and oral health status, examining microbial diversity and the prevalence of specific bacterial genera. We conducted a cross-sectional study of 154 patients who underwent oral examinations at our hospital from January 2023 to December 2023. Based on routine oral examinations and medical history inquiries, patients were divided into oral health group (n = 71) and oral health disease group (n = 83). Oral samples were collected and analyzed using next-generation sequencing and bioinformatics to assess microbial diversity and abundance. Key demographic and behavioral factors were recorded, and statistical analyses determined oral microbiome characteristics with oral health. The oral health disease group had significantly higher average age, smoking rates, body mass index, alcohol use, family history of oral diseases, and worse oral health indicators (e.g., plaque, gum inflammation, calculus, periodontal status, decayed/missing teeth, and decayed, missing, and filled teeth scores). They also showed lower microbial diversity (Shannon, Chao1, and evenness indices) and reduced levels of protective bacteria (*Streptococcus* and *Actinomyces*). In contrast, the oral health group had higher flossing rates, more caries-free individuals, and greater microbial diversity. The Shannon, Chao1, and evenness indices were inversely linked to oral disease, while the Simpson Index (measuring dominance) was positively correlated. Protective bacteria were negatively linked to disease, whereas harmful bacteria (*Fusobacterium* and *Prevotella*) were positively correlated. Our findings highlight decreased microbial diversity as a significant factor in oral diseases, suggesting that maintaining a diverse oral microbiome was crucial for oral health.

## 1. Introduction

Oral health was a critical component of general health and quality of life, significantly affecting social, psychological, and medical outcomes.^[1]^ Despite advances in oral healthcare, oral diseases such as dental caries and periodontal diseases remain prevalent global health concerns, affecting millions of people worldwide.^[2–4]^ These conditions were influenced by complex interactions between the host, environmental factors, and the oral biofilms.^[5]^ The development of oral diseases has been increasingly linked to changes in the oral microbiome, an intricate ecosystem composed of diverse microorganisms that reside predominantly in the oral cavity.^[6–8]^

Research has established that the oral cavity was home to more than 700 bacterial species, forming complex biofilms that contribute to maintaining the balance between health and disease.^[9]^ In a healthy state, the oral microbiome operates symbiotically, engaging in protective roles against pathogenic species and contributing to the host’s immune defenses. However, disturbances in this microbial equilibrium, often referred to as dysbiosis, have been implicated in the pathogenesis of various oral diseases.^[10,11]^ Dysbiosis can emerge from multiple factors, including poor oral hygiene, smoking, alcohol consumption, diet, and underlying systemic health conditions, leading to an overgrowth of pathogenic bacteria and diminishing microbial diversity.^[12]^

Understanding the oral microbiome’s role in health and disease necessitates a thorough exploration of microbial diversity and abundance. Recent advances in molecular biology techniques, such as next-generation sequencing, have facilitated comprehensive analyses of oral microbiota, highlighting the importance of microbial diversity as a marker of oral health.^[13]^ Notably, a diverse and stable microbiome was often associated with health, capable of resisting disease-associated bacteria through mechanisms like competitive exclusion and immune modulation. Conversely, a decrease in this diversity may contribute to the predominance of pathogenic communities, fostering environments conducive to oral diseases.

Previous studies have identified specific microbial patterns associated with oral health conditions. For instance, an increase in pathogens such as *Fusobacterium* and *Prevotella* was commonly observed in oral diseases, particularly periodontitis, which was characterized by inflamed and receding gums, along with the destruction of supporting structures of the teeth.^[14]^ These bacteria were known to produce virulence factors that exacerbate epithelial and connective tissue disruption, leading to disease progression.^[15]^ On the other hand, beneficial genera like *Streptococcus* and *Actinomyces* were often found in greater abundance in oral health, contributing to a balanced microbial ecosystem. These genera exert their beneficial effects through producing bacteriocins that inhibit the growth of pathogenic species and modulating host immune responses. The research method involves amplifying and sequencing the bacterial 16S ribosomal ribonucleic acid gene from oral samples (such as saliva and dental plaque) to analyze the composition and diversity of the bacterial community.

Despite these insights, the complex interconnections among microbial diversity, abundance, lifestyle attributes, and oral health remain incompletely understood. Comprehensive analyses that integrate microbial, demographic, and behavioral data were essential to delineate these relationships more clearly.

This study aimed to elucidate the correlations between oral microbiome characteristics and oral health conditions by examining microbial diversity indices and the relative abundance of key bacterial genera in relation to demographic and behavioral factors.

## 2. Materials and methods

### 2.1. Case selection

This study was approved by the Ethics Committee of Guangdong Provincial People’s Hospital’s Nanhai Hospital. The study conducted was a retrospective cross-sectional analysis incorporating 154 patients who underwent oral examinations at our hospital from January 2023 to December 2023. The oral health and oral disease groups were defined according to the World Health Organization (WHO) international standard. The oral health standard is: clean teeth, no caries, no pain, normal gingival color, no bleeding, if abnormal, they were divided into oral disease group.

Demographic information was collected from patient records, including general patient data, oral hygiene index, bacterial diversity index, and predominance index. Given that this retrospective study utilized only de-identified patient data, there was no potential risk or impact on the medical care of the patients. Oral swab samples were collected during routine examinations and archived for potential research. Ethics approval covered both initial sample collection and retrospective data analysis (Approval NO. 2023216H). Consequently, informed consent was waived. This waiver and the study itself received approval from both the hospital’s ethics review committee and the ethics committee, in accordance with regulatory and ethical guidelines for retrospective studies.

### 2.2. Inclusion and exclusion criteria

To ensure standardized and reproducible classification, the following objective criteria were strictly applied by 2 calibrated dentists (with ≥ 5 years of clinical experience) using WHO-approved tools:

1. “Clean teeth”: plaque index (PI) ≤ 1 at all examined sites (Quigley-Hein index modified by Turesky).^[16]^
2. “No caries”: Absence of cavitated lesions (International Caries Detection and Assessment System [ICDAS] score 0–2), verified via visual-tactile examination with a WHO probe under artificial light.^[17]^
3. “No pain”: patient-reported absence of spontaneous or evoked pain during examination.
4. “Normal gingival color”: gingival index (GI) = 0 (pale pink) with no erythema or edema.^[18]^
5. “No bleeding”: bleeding on probing (BOP) ≤ 10% of sites.^[19]^

Inter-examiner calibration was performed prior to the study (Kappa = 0.85 for caries diagnosis; intraclass correlation coefficient > 0.8 for indices). Disagreements were resolved by a third senior periodontist.

Inclusion criteria: patient’s clinical symptoms and diagnosis meet the criteria for oral disease diagnosis^[20,21]^; individuals exhibiting normal mental and cognitive functions; individuals with complete medical records; individuals aged over 18 years.

Exclusion criteria: patients diagnosed with cirrhosis or malignant tumors; individuals with severe immune disorders; patients with a history of antibiotic use, immune preparation, hormone medication, or microbiota regulators within the past month. Diagnoses of cirrhosis, malignancies, severe immune disorders, and recent medication use were verified through electronic health records, including physician notes, laboratory results, radiology reports, and pharmacy data.

**Figure 1.**
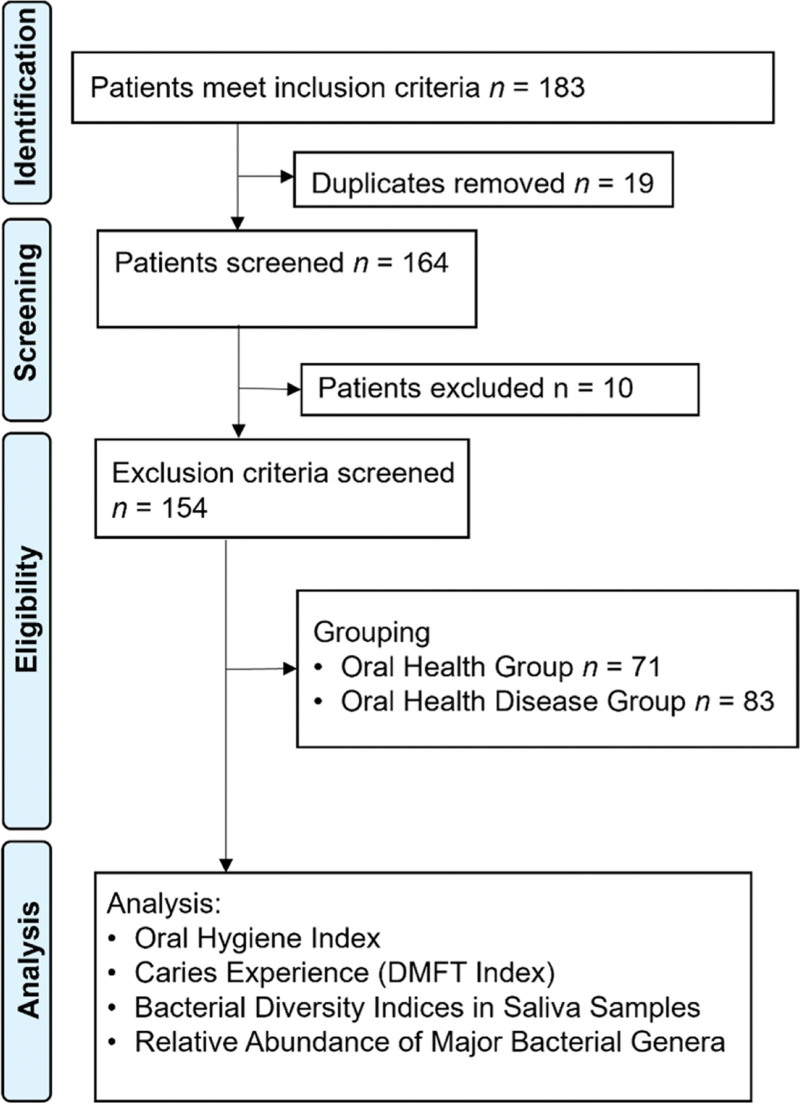
Patient selection flowchart. DMFT = decayed, missing, filled teeth, n= number of patients.

The patient selection flowchart is shown in Figure 1.

### 2.3. Oral examination process

The patient was seated in a chair for an oral examination conducted under artificial lighting, employing disinfected tools such as intraoral mirrors, WHO probes, and disposable gloves. The oral health status was recorded in detail by dentists from our hospital based on routine oral examinations and medical history inquiries. Examinations were conducted independently by 2 pre-calibrated dentists using sterilized WHO probes, mouth mirrors, and periodontal probes under standardized illumination. Diagnostic criteria adhered to:

1. Caries: ICDAS-II criteria.^[16]^
2. Periodontal health: community periodontal index (CPI) = 0 (no pockets > 3 mm, BOP < 10%), GI ≤ 1 (Löe-Silness GI).^[22]^
3. Oral mucosal lesions: WHO guide to epidemiology and diagnosis of oral mucosal diseases.^[23]^

All examiners completed a 4-hour training module on WHO criteria and achieved > 90% agreement with gold-standard diagnoses during calibration. Data were cross-validated for consistency. Within the oral health disease group (n = 83), conditions were categorized as: plaque/calculus-induced gingivitis (n = 10): BOP > 10%, GI ≥ 1, CPI = 1 to 2^[19,22]^; Periodontitis (n = 8): CPI ≥ 3, radiographic bone loss^[20]^; Dental caries (n = 14): ICDAS ≥ 3^[17]^; Calculus with inflammation (n = 27): calculus index [CI]> 1, GI ≥ 1; Tooth loss due to caries/periodontitis (n = 22); Oral mucosal lesions (n = 2). All conditions involved active disease with clinical signs of inflammation or tissue destruction. The oral health group (n = 71) was defined as patients who did not present with any oral diseases. The Turesky-Gilmore-Glickman modification of the Quigley-Hein PI, GI, and dentition status and treatment needs at baseline, and at the third and sixth months of the study period were recorded. The decayed, missing, and filled teeth (DMFT) index was calculated based on the dentition status and treatment needs. Indices (PI, GI, DMFT) were recorded at the baseline examination only, consistent with the cross-sectional design.

### 2.4. Microbial sampling

Prior to sample collection, the patient refrained from eating, smoking, or consuming alcohol for at least 1 hour to ensure no contamination of the oral cavity. The patient then thoroughly rinsed their mouth with clean water 2 to 3 times to remove any foreign objects. To collect the sample, the outer packaging of the cotton swab was opened, and the swab was held by the handle for insertion into the oral cavity. The tip of the swab was gently pressed against the inside of the cheek, applying enough force to create a slight convexity. The swab was then moved up and down while being simultaneously rotated, with twenty scrapes performed per cheek. Samples were collected from both the left and right cheeks. Upon completion, the swab was returned to the collection tube, which was labeled with the patient’s name. Relevant patient information, including name, gender, age, collection date, and group information, was registered on the label. The collected oral swabs were subsequently stored in a −80°C freezer.

### 2.5. Oral colony deoxyribonucleic acid (DNA) extraction

Bacterial community profiling was performed through 16S ribosomal ribonucleic acid gene sequencing. The V3-V4 hypervariable regions were amplified using primers 341F (5’-CCTACGGGNGGCWGCAG-3’) and 805R (5’-GACTACHVGGGTATCTAATCC-3’). Libraries were prepared with dual-indexed barcodes and sequenced on the Illumina NovaSeq 6000 platform (2 × 250 bp paired end). Bacterial DNA was extracted using the cetyltrimethylammonium bromide method. Subsequently, the DNA samples were amplified via polymerase chain reaction. A 2% agarose gel prepared with 1 × Tris-acetate-EDTA buffer (G2151-1L, Servicebio, Wuhan, Hubei, China) was used to purify the polymerase chain reaction products, which were then recovered using the GeneJET Gel Recovery Kit (K0702, Thermo Fisher Scientific Inc., Waltham). Libraries were constructed using the Ion Plus Fragment Library Kit for 48 reactions. The constructed libraries underwent quantification and detection using Qubit, followed by sequencing with Ion S5TM XL. Low-abundance taxa were filtered using a 0.005% minimum relative abundance threshold across all samples. Operational taxonomic units (OTUs) present in fewer than 5% of samples were excluded to minimize spurious signals. For relative abundance analysis, data underwent centered log-ratio transformation after adding a pseudo-count of 0.5 to handle zeros. Alpha diversity metrics (Shannon, Simpson, Chao1) were calculated from rarefied OTU tables normalized to 18,000 sequences per sample. Raw data were processed by trimming and filtering to obtain valid data, which were then clustered into OTUs. These OTUs were selected as representative sequences and compared against the SILVA132 database for species annotation. Bioinformatics techniques were applied for alpha diversity analysis to verify the adequacy of the sequencing depth and to gain an initial understanding of species diversity and abundance. Principal coordinates analysis, a beta diversity analysis, was conducted to explore differences in species structure between groups. Significant differences in species between groups were identified using LEfSe analysis. Finally, normalization was performed on the data from all samples, and the results were analyzed, including the Chao1 index, Shannon index, and Simpson index.

### 2.6. Statistical method

Using G*Power 3.1.9.7, based on the “*t*-tests: means - Difference between 2 independent means (two groups)” option, select post hoc analysis, set: Two-tailed mode, Effect size d = 0.5, α err prob = 0.05, then enter the sample sizes of the 2 groups, and calculate Power (1-β err prob), with a result of 0.867.

Data analysis was conducted using SPSS version 29.0 (SPSS Inc., Chicago, IL). Categorical variables were expressed as [n (%)]. For sample sizes of ≥ 40 with an expected frequency T ≥ 5, the chi-square test was performed using the standard formula. When the sample size was ≥ 40 but with an expected frequency 1 ≤ T < 5, the corrected formula for the chi-square test was applied. For sample sizes < 40 or when the expected frequency was T < 1, Fisher exact test was utilized for statistical analysis. The Shapiro–Wilk method was employed to test the normality of continuous variables. Continuous variables following a normal distribution were expressed as (mean ± standard deviation) and analyzed using the *t*-test with corrected variance. Non-normally distributed continuous variables are presented as medians (25th percentile, 75th percentile) and analyzed using the Wilcoxon rank-sum test. A 2-sided *P* value of < .05 was considered statistically significant. Pearson correlation analysis was used to examine the relationship between the characteristics of the oral microbiome and oral health diseases.

## 3. Results

### 3.1. Demographic characteristics

Of the 83 patients with oral health diseases, 27 had dental calculus, 22 had missing teeth, 14 had dental caries, 8 had periodontitis, 10 had gingivitis, and 2 had oral mucosal lesions. The average age (50.34 ± 13.44 years vs 45.23 ± 12.89 years; *P* = .018), smoking rate (34.94% vs 19.72%; *P* = .036), body mass index (BMI) (25.56 ± 3.89 kg/m^2^ vs 23.78 ± 3.12 kg/m^2^; *P* = .002), alcohol consumption rate (43.37% vs 26.76%; *P* = .032), and family history of oral diseases (45.78% vs 29.58%; *P* = .039) in the oral health disease group were significantly higher than those in the oral health group, while the use of dental floss(50.70% vs 30.12%; *P* = .009) was significantly lower in the oral health disease group compared to the oral health group (Table 1). Gender distribution showed no significant difference (*P* = .756). Educational level differences were significant, favoring higher education in the health group (*P* = .030). The frequency of daily brushing was found to be statistically less frequent in the Oral Health Disease Group (*P* = .011). These findings suggest significant correlations between several behavioral and demographic factors with oral health status.

**Table 1:** Demographic characteristics of study participants.

Parameters	Oral Health Group (n = 71)	Oral Health Disease Group (n = 83)	*t*/*χ*^2^	*P*
Age (yr)	45.23 ± 12.89	50.34 ± 13.44	2.397	.018
Gender (male/female)	35 (49.30%)/36 (50.70%)	43 (51.81%)/40 (48.19%)	0.097	.756
Smoking habits	14 (19.72)	29 (34.94)	4.405	.036
BMI (kg/m^2^)	23.78 ± 3.12	25.56 ± 3.89	3.113	.002
Educational level (n, %)			6.998	.030
Primary	7 (9.86)	17 (20.48)		
Secondary	30 (42.25)	42 (50.60)		
Higher	34 (47.89)	24 (28.92)		
Alcohol consumption	19 (26.76)	36 (43.37)	4.600	.032
Daily brushing frequency (n, %)			8.978	.011
Once	4 (5.63)/	18 (21.69)		
Twice	50 (70.42)	53 (63.86)		
More than twice	17 (23.94)	12 (14.46)		
Use of dental floss	36 (50.70)	25 (30.12)	6.778	.009
Family history of oral disease	21 (29.58)	38 (45.78)	4.252	.039

BMI = body mass index, n = number of participants.

### 3.2. Oral hygiene index and caries experience

The PI was significantly higher in the Oral Health Disease Group (1.21 ± 0.46) compared to the Oral Health Group (1.05 ± 0.42; *P* = .027) (Table 2). Similarly, the GI showed a significantly higher value in the disease group (1.12 ± 0.31) versus the health group (0.97 ± 0.28; *P* = .001). The CI also indicated a significant elevation in the Oral Health Disease Group (0.83 ± 0.34) relative to the Oral Health Group (0.69 ± 0.22; *P* = .003). Additionally, the CPI was notably higher in the disease group (1.24 ± 0.57) compared to the health group (1.02 ± 0.51; *P* = .013). Finally, BOP was more prevalent in the Oral Health Disease Group, reflected by a higher percentage (16.96 ± 5.78%) compared to the Oral Health Group (15.14 ± 5.43%; *P* = .047). Individuals in the disease group had a higher mean number of decayed teeth (1.78 ± 0.89) compared to the health group (1.45 ± 0.23; *P* = .002). Similarly, the mean number of missing teeth was significantly higher in the Oral Health Disease Group (2.03 ± 0.45) versus the Oral Health Group (1.86 ± 0.34; *P* = .005). The filled tooth count also showed a significant difference, with the disease group having 3.26 ± 1.33 filled teeth compared to 2.56 ± 1.29 in the health group (*P* = .001). Consequently, the total DMFT Index was significantly elevated in the disease group (5.01 ± 1.32) compared to the health group (4.36 ± 1.12; *P* = .001). Furthermore, the proportion of caries-free individuals was significantly lower in the Oral Health Disease Group (38.55%) than in the Oral Health Group (57.75%; *P* = .017). These results underscore significant associations between poor oral hygiene indices and increased caries experience and the presence of oral health disease.

**Table 2:** Oral hygiene index and caries experience.

Parameters	Oral Health Group (n = 71)	Oral Health Disease Group (n = 83)	*t*	*P*
PI	1.05 ± 0.42	1.21 ± 0.46	2.237	.027
GI	0.97 ± 0.28	1.12 ± 0.31	3.252	.001
CI	0.69 ± 0.22	0.83 ± 0.34	3.075	.003
CPI	1.02 ± 0.51	1.24 ± 0.57	2.517	.013
BOP (%)	15.14 ± 5.43	16.96 ± 5.78	2.005	.047
Caries Experience (DMFT Index)				
Decayed (D)	1.45 ± 0.23	1.78 ± 0.89	3.257	.002
Missing (M)	1.86 ± 0.34	2.03 ± 0.45	2.834	.005
Filled (F)	2.56 ± 1.29	3.26 ± 1.33	3.301	.001
DMFT Total	4.36 ± 1.12	5.01 ± 1.32	3.242	.001
Number of Caries-Free (n, %)	41 (57.75)	32 (38.55)	5.653	.017

BOP = bleeding on probing, CI = calculus index, CPI = community periodontal index, DMFT = decayed, missing, filled teeth, GI = gingival index, n = number of participants, PI = plaque index.

### 3.3. Bacterial diversity indices and relative abundance

The Shannon Index, indicative of microbial diversity, was significantly lower in the Oral Health Disease Group (2.10 ± 0.56) compared to the Oral Health Group (2.35 ± 0.44; *P* = .002) (Table 3). The Simpson Index, which reflects diversity dominance, was higher in the disease group (0.25 ± 0.07) than in the health group (0.22 ± 0.06; *P* = .004), indicating less evenness in microbial distribution. Chao1 Index, a measure of estimated species richness, was reduced in the disease group (127.78 ± 17.55) relative to the health group (134.34 ± 15.23; *P* = .015). Microbial Evenness was significantly reduced in the disease group (0.75 ± 0.13) compared to the health group (0.81 ± 0.17; *P* = .020). The relative abundance of *Streptococcus* was significantly lower in the Oral Health Disease Group (22.42 ± 4.98%) compared to the Oral Health Group (24.79 ± 5.23%; *P* = .005). Similarly, *Actinomyces* was less abundant in the disease group (5.56 ± 1.45%) than in the health group (6.34 ± 1.67%; *P* = .002). Conversely, *Fusobacterium* and *Prevotella* showed higher relative abundance in the disease group, with *Fusobacterium* at 8.17 ± 3.23% versus 7.12 ± 2.43% in the health group (*P* = .023), and *Prevotella* at 8.78 ± 3.49% compared to 7.54 ± 2.74% (*P* = .015). The difference in the relative abundance of *Veillonella* between the 2 groups was not statistically significant (*P* = .369). These results underscore a clear association between microbial diversity of individuals and relative abundance and oral health disease.

**Table 3:** Bacterial diversity indices and relative abundance.

Parameters	Oral Health Group (n = 71)	Oral Health Disease Group (n = 83)	*t*	*P*
Shannon Index	2.35 ± 0.44	2.10 ± 0.56	3.088	.002
Simpson Index	0.22 ± 0.06	0.25 ± 0.07	2.885	.004
Chao1 Index	134.34 ± 15.23	127.78 ± 17.55	2.454	.015
Evenness	0.81 ± 0.17	0.75 ± 0.13	2.352	.020
*Streptococcus* (%)	24.79 ± 5.23	22.42 ± 4.98	2.872	.005
*Actinomyces* (%)	6.34 ± 1.67	5.56 ± 1.45	3.102	.002
*Fusobacterium* (%)	7.12 ± 2.43	8.17 ± 3.23	2.290	.023
*Prevotella* (%)	7.54 ± 2.74	8.78 ± 3.49	2.467	.015
*Veillonella* (%)	9.67 ± 3.12	9.23 ± 2.89	0.901	.369

n = number of participants.

### 3.4. Multivariate logistic regression

After adjusting for age, smoking habits, alcohol consumption, BMI, educational level, daily brushing frequency, use of dental floss, and family history of oral disease, Chao1 richness, observed. Species, *Streptococcus*, *Actinomyces*, *Fusobacterium* and *Prevotella* showed a significant association with oral health disease, with odds ratio value at 0.97 (95% CI 0.94–0.99, *P* = .009), 0.97 (95%, 0.94–1, *P* = .036), 0.91 (95% CI 0.84–0.98, *P* = .011), 0.77 (95% CI 0.6–1, *P* = .047), 1.15 (95% CI 1–1.32, *P* = .044), and 1.15 (95% CI 1.02–1.3, *P* = .027) respectively (Table 4).

**Table 4:** Multivariate logistic regression.

Oral Health Diseas Parameters	Crude OR (95% CI)*	*P* value	OR (95% CI)†	*P* value
Shannon Index	0.37 (0.19–0.73)	.004	0.47 (0.21–1.07)	.071
Simpson Index	95.94 (1.18–7804.12)	.042	137.23 (0.76–24,750.51)	.063
Chao1 Richness	0.98 (0.96–1)	.017	0.97 (0.94–0.99)	.009
Observed Species	0.98 (0.95–1)	.039	0.97 (0.94–1)	.036
Evenness	0.08 (0.01–0.75)	.026	0.1 (0.01–1.43)	.09
*Streptococcus*	0.91 (0.85–0.97)	.006	0.91 (0.84–0.98)	.011
*Actinomyces*	0.72 (0.58–0.9)	.003	0.77 (0.6–1)	.047
*Fusobacterium*	1.14 (1.01–1.27)	.029	1.15 (1–1.32)	.044
*Prevotella*	1.13 (1.02–1.26)	.019	1.15 (1.02–1.3)	.027

BMI = body mass index, CI = calculus index, n = number of participants, OR = odds ratio.

* Crude model.

† Adjusted for age, smoking habits, alcohol consumption, BMI, educational level, daily brushing frequency, use of dental floss, family history of oral disease.

## 4. Discussion

Firstly, our analysis highlighted a decreased microbial diversity in the Oral Health Disease Group compared to the Oral Health Group, as evidenced by the significantly lower Shannon Index and Chao1 Index. This reduced diversity appears to be a pivotal feature associated with oral diseases, supporting the hypothesis that a balanced and diverse microflora was crucial for maintaining oral health. Mechanistically, a diverse microbiome can prevent overgrowth of pathogenic bacteria by competitive exclusion and can enhance immune modulation.^[24]^ When diversity wanes, niche spaces previously occupied by beneficial commensals may be taken over by opportunistic pathogens such as *Fusobacterium* and *Prevotella*, which were found in greater abundance in the disease group. These bacteria were known to be involved in the pathogenesis of periodontal diseases and can undermine tissue integrity through their virulence factors, promoting inflammation and tissue destruction.^[25,26]^

The study also revealed that age, smoking habits, BMI, family history of oral disease, educational level, frequent tooth brushing, and use of dental floss were significant different between the 2 groups. From a mechanistic perspective, aging was accompanied by physiological changes that predispose individuals to chronic diseases, including oral conditions. The natural decline in immune function and changes in salivary composition with age could lead to a disrupted oral microbiome, thus facilitating disease.^[27]^ Similarly, smoking introduces numerous noxious chemicals into the oral cavity, which not only promote inflammation and suppress immune responses but also encourage the growth of certain pathogenic bacterial species. Smoking may also interfere with the microbiome’s ability to metabolize certain substrates, thus altering the balance of microbial communities.^[28,29]^

The relationship between elevated BMI and oral disease could be explained by shared underlying systemic inflammation and metabolic disturbances that promote both obesity and periodontal disease. Obesity was marked by chronic inflammatory states and dysbiosis, which can similarly affect the oral cavity.^[30]^ These insights align with broader public health findings that suggest systemic health conditions can mirror oral health profiles.

Educational level and oral hygiene practices such as brushing frequency and flossing were significantly protective against oral diseases, reflecting the critical role of informed and active maintenance of oral hygiene. Regular removal of food particles and dental plaque can reduce the substrate availability for bacterial growth, thereby curtailing the proliferation of harmful bacteria and maintaining a balanced oral microbiome.^[31,32]^ This finding emphasizes the importance of public health initiatives aimed at improving educational resources and emphasizing oral hygiene. Interestingly, the study also highlighted an inverse correlation between *Streptococcus* and *Actinomyces* levels and oral disease presence. These genera, commonly associated with health, tend to contribute positively to microfloral homeostasis, possibly through the production of bacteriocins that suppress pathogenic species or by priming the host immune system for balanced responses. Their reduced presence in the disease group suggests that strategies aimed at rebalancing oral microbiota through prebiotics or targeted probiotics could be beneficial.

Moreover, our findings suggested significant differences in oral hygiene indices such as the PI, GI, and CI between the 2 groups. Elevated indices in the disease group underscore the contribution of poor oral hygiene to disease, likely due to increased bacterial biofilm formation that can harbor pathogenic species. This biofilm acts as a reservoir for bacteria that induce gingival inflammation and periodontal breakdown, a pathway well-documented in periodontal disease pathogenesis.^[7,33]^

The varying abundances of specific genera such as *Fusobacterium* and *Prevotella* may also interplay with inflammatory responses and tissue health. These bacteria often co-aggregate with each other and with other pathogenic bacteria in the biofilm, facilitating a conducive environment for disease progression through synergistic interactions where metabolic byproducts of 1 species serve as nutrients for others.^[34]^

Caries experience, measured through the DMFT Index, was markedly higher in the disease group, suggesting that carious lesions both influence and result from microbiome alterations. As dental caries progresses, the environment becomes more conducive to acidogenic and aciduric bacteria, which further deteriorates the hard tissues of teeth and possibly alters the adjacent microbial composition.^[35,36]^ The correlation between caries experience and oral disease aligns with comprehensive preventive approaches that must address interrelated factors, including diet, fluoride exposure, and regular dental checks to reduce caries and associated bacterial shifts.^[2,37]^

While this study provides valuable insights into the correlation between oral microbiome characteristics and oral health conditions, several limitations must be acknowledged. Firstly, the study’s retrospective, single-center design inherently limits the ability to establish causative relationships, as it primarily reveals associations, and may introduce selection bias that could affect the generalizability of the findings to broader populations with differing demographic and socio-economic backgrounds. Furthermore, the reliance on de-identified data may have constrained our ability to account for all potential confounding variables. Notably, dietary factors (such as sugar intake and eating patterns) which are well-established contributors to both oral microbiome composition and oral disease risk, were not captured in this study. Additionally, while we excluded patients with a history of antibiotic use within the past month, the relatively short recall period may not fully account for the potential longer-term effects of antibiotic exposure on the oral microbiome. Other unmeasured confounders, such as genetic predispositions to oral diseases, may also exist. Moreover, while the microbial analyses provided rich data on bacterial abundance and diversity, they were limited to specific bacteria and did not encompass the full spectrum of the oral microbiome, including fungi and viruses, which might also play significant roles in oral health.^[38]^ Finally, the cross-sectional nature of the microbiome analysis does not capture temporal changes in microbial communities that might be critical in understanding disease progression. Future studies should aim to incorporate longitudinal designs, include multicenter cohorts to enhance generalizability, and expand the scope of microbiome profiling for a more comprehensive understanding.

## 5. Conclusion

In conclusion, our study reveals significant associations between the oral microbiome, modifiable lifestyle factors, and oral health status. The observed decline in microbial diversity, along with shifts in the abundance of specific bacterial genera, appears to be closely linked to the presence of oral diseases. These findings suggest potential avenues for further investigation, such as exploring whether microbiome modulation through lifestyle changes, dietary adjustments, or bacteriotherapy may contribute to oral health maintenance. Our results highlight the importance of a holistic approach to oral healthcare that considers microbial ecology alongside traditional hygiene practices. Further research, particularly longitudinal studies, is warranted to better understand the temporal dynamics of these relationships and to explore strategies for microbiome preservation and restoration in comprehensive oral health management.

## Author contributions

**Conceptualization:** Yujie Nie, Xiaohui Lin.

**Data curation:** Yujie Nie, Xiaohui Lin.

**Formal analysis:** Yujie Nie, Xiaohui Lin.

**Funding acquisition:** Yujie Nie.

**Investigation:** Yujie Nie.

**Writing – original draft:** Yujie Nie, Xiaohui Lin.

**Writing – review & editing:** Yujie Nie, Xiaohui Lin.

## References

[1] GBD 2021 Stroke Risk Factor Collaborators. Global, regional, and national burden of stroke and its risk factors, 1990-2021: a systematic analysis for the Global Burden of Disease Study 2021. Lancet Neurol. 2024;23:973–1003. 39304265 10.1016/S1474-4422(24)00369-7 PMC12254192

[2] Al-Nasser L Lamster IB. Prevention and management of periodontal diseases and dental caries in the older adults. Periodontology 2000. 2020;84:69–83. 32844424 10.1111/prd.12338

[3] Banihashem Rad SA Esteves-Oliveira M Maklennan A Douglas GVA Castiglia P Campus G. Oral health inequalities in immigrant populations worldwide: a scoping review of dental caries and periodontal disease prevalence. BMC Public Health. 2024;24:1968. 39044172 10.1186/s12889-024-19354-4 PMC11267954

[4] Benzian H Listl S. [Global oral health in the international health policy spotlight-challenges and new opportunities for sustainable improvement]. Bundesgesundheitsblatt, Gesundheitsforschung, Gesundheitsschutz. 2021;64:871–8. 34100957 10.1007/s00103-021-03353-6 PMC8185487

[5] Sedghi L DiMassa V Harrington A Lynch SV Kapila YL. The oral microbiome: Role of key organisms and complex networks in oral health and disease. Periodontology 2000. 2021;87:107–31. 34463991 10.1111/prd.12393 PMC8457218

[6] Curtis MA Diaz PI Van Dyke TE. The role of the microbiota in periodontal disease. Periodontology 2000. 2020;83:14–25. 32385883 10.1111/prd.12296

[7] Di Stefano M Polizzi A Santonocito S Romano A Lombardi T Isola G. Impact of oral microbiome in periodontal health and periodontitis: a critical review on prevention and treatment. Int J Mol Sci. 2022;23:5142. 35563531 10.3390/ijms23095142 PMC9103139

[8] Pathak JL Yan Y Zhang Q Wang L Ge L. The role of oral microbiome in respiratory health and diseases. Respir Med. 2021;185:106475. 34049183 10.1016/j.rmed.2021.106475

[9] Mosaddad SA Tahmasebi E Yazdanian A. Oral microbial biofilms: an update. Eur J Clin Microbiol Infect Dis. 2019;38:2005–19. 31372904 10.1007/s10096-019-03641-9

[10] Hou K Wu ZX Chen XY. Microbiota in health and diseases. Signal Transduct Target Ther. 2022;7:135. 35461318 10.1038/s41392-022-00974-4 PMC9034083

[11] Peng X Cheng L You Y. Oral microbiota in human systematic diseases. Int J Oral Sci. 2022;14:14. 35236828 10.1038/s41368-022-00163-7 PMC8891310

[12] Santacroce L Passarelli PC Azzolino D. Oral microbiota in human health and disease: a perspective. Exp Biol Med (Maywood). 2023;248:1288–301. 37688509 10.1177/15353702231187645 PMC10625343

[13] Grier A Myers JA O’Connor TG Quivey RG Gill SR Kopycka-Kedzierawski DT. Oral microbiota composition predicts early childhood caries onset. J Dent Res. 2021;100:599–607. 33356775 10.1177/0022034520979926 PMC8142088

[14] Lima KM Alves CM Vidal FC. Fusobacterium nucleatum and prevotella in women with periodontitis and preterm birth. Med Oral Patol Oral Cir Bucal. 2023;28:e450–6. 37622431 10.4317/medoral.25874 PMC10499342

[15] Elnar AG Kim GB. In vitro and in silico characterization of n-formylated two-peptide bacteriocin from enterococcus faecalis CAUM157 with anti-listeria activity. Probiotics Antimicrob Proteins. 2024;16:1130–47. 38743207 10.1007/s12602-024-10265-9

[16] Turesky S Gilmore ND Glickman I. Reduced plaque formation by the chloromethyl analogue of 3,4,5-trimethoxyphenylthiohydantoin. J Periodontol. 1970;41:41–3. 5264376 10.1902/jop.1970.41.41.41

[17] Ismail AI Sohn W Tellez M. The international caries detection and assessment system (ICDAS): an integrated system for measuring dental caries. Community Dent Oral Epidemiol. 2007;35:170–8. 17518963 10.1111/j.1600-0528.2007.00347.x

[18] Lobene RR Weatherford T Ross NM Lamm RA Menaker L. A modified gingival index for use in clinical trials. Clin Prev Dent. 1986;8:3–6. 3485495

[19] Page RC Eke PI. Case definitions for use in population-based surveillance of periodontitis. J Periodontol. 2007;78(7 suppl):1387–99. 10.1902/jop.2007.060264 17608611

[20] Duncan HF Kirkevang LL Peters OA. Treatment of pulpal and apical disease: the european society of endodontology (ESE) S3-level clinical practice guideline. Int Endod J. 2023;56(Suppl 3):238–95. 37772327 10.1111/iej.13974

[21] Sanz M Herrera D Kebschull M. Treatment of stage I-III periodontitis-The EFP S3 level clinical practice guideline. J Clin Periodontol. 2020;47(Suppl 22):4–60. 32383274 10.1111/jcpe.13290 PMC7891343

[22] Ainamo J Barmes D Beagrie G Cutress T Martin J Sardo-Infirri J. Development of the World Health Organization (WHO) community periodontal index of treatment needs (CPITN). Int Dent J. 1982;32:281–91. 6958657

[23] World Health Organization. Oral Health Surveys: Basic Methods. 5th ed. World Health Organization; 2013.

[24] Forgie AJ Pepin DM Ju T. Over supplementation with vitamin B12 alters microbe-host interactions in the gut leading to accelerated Citrobacter rodentium colonization and pathogenesis in mice. Microbiome. 2023;11:21. 36737826 10.1186/s40168-023-01461-w PMC9896722

[25] Du Q Ma X. [Research progress of correlation between periodontal pathogens and systemic diseases]. Nan Fang Yi Ke Da Xue Xue Bao. 2020;40:759–64. 32897213 10.12122/j.issn.1673-4254.2020.05.24 PMC7277321

[26] Gönczi NN Strang O Bagi Z Rákhely G Kovács KL. Interactions between probiotic and oral pathogenic strains. Biol Futur. 2021;72:461–71. 34554489 10.1007/s42977-021-00091-3

[27] Liu Y Wang X Jin C. Total ginsenosides extend healthspan of aging Drosophila by suppressing imbalances in intestinal stem cells and microbiota. Phytomedicine. 2024;129:155650. 38669971 10.1016/j.phymed.2024.155650

[28] Chaffee BW Couch ET Vora MV Holliday RS. Oral and periodontal implications of tobacco and nicotine products. Periodontol 2000. 2021;87:241–53. 34463989 10.1111/prd.12395 PMC8444622

[29] Gormley M Dudding T Sanderson E. A multivariable Mendelian randomization analysis investigating smoking and alcohol consumption in oral and oropharyngeal cancer. Nat Commun. 2020;11:6071. 33247085 10.1038/s41467-020-19822-6 PMC7695733

[30] Kuraji R Sekino S Kapila Y Numabe Y. Periodontal disease-related nonalcoholic fatty liver disease and nonalcoholic steatohepatitis: An emerging concept of oral-liver axis. Periodontol 2000. 2021;87:204–40. 34463983 10.1111/prd.12387 PMC8456799

[31] Dudek-Wicher R Junka AF Migdał P Korzeniowska-Kowal A Wzorek A Bartoszewicz M. The antibiofilm activity of selected substances used in oral health prophylaxis. BMC Oral Health. 2022;22:509. 36397044 10.1186/s12903-022-02532-4 PMC9672622

[32] La X Jiang H Chen A. Profile of the oral microbiota from preconception to the third trimester of pregnancy and its association with oral hygiene practices. J Oral Microbiol. 2022;14:2053389. 35341210 10.1080/20002297.2022.2053389 PMC8942530

[33] Kedlaya MN Puzhankara L Prasad R Raj A. Periodontal disease pathogens, pathogenesis, and therapeutics: the CRISPR-Cas effect. CRISPR J. 2023;6:90–8. 36939849 10.1089/crispr.2022.0094

[34] Jakubovics NS Goodman SD Mashburn-Warren L Stafford GP Cieplik F. The dental plaque biofilm matrix. Periodontol 2000. 2021;86:32–56. 33690911 10.1111/prd.12361 PMC9413593

[35] Shao Q Feng D Yu Z. The role of microbial interactions in dental caries: dental plaque microbiota analysis. Microb Pathog. 2023;185:106390. 37858633 10.1016/j.micpath.2023.106390

[36] Spatafora G Li Y He X Cowan A Tanner ACR. The evolving microbiome of dental caries. Microorganisms. 2024;12:121. 38257948 10.3390/microorganisms12010121 PMC10819217

[37] Soares RC da Rosa SV Moysés ST. Methods for prevention of early childhood caries: overview of systematic reviews. Int J Paediatr Dent. 2021;31:394–421. 33263186 10.1111/ipd.12766

[38] Krumbeck JA Reiter AM Pohl JC. Characterization of oral microbiota in cats: novel insights on the potential role of fungi in feline chronic gingivostomatitis. Pathogens. 2021;10:904. 34358054 10.3390/pathogens10070904 PMC8308807

